# Parameter optimization analysis of rotary electromagnetic vibration energy harvester for performance enhancement under free vibration

**DOI:** 10.1016/j.isci.2023.107989

**Published:** 2023-09-21

**Authors:** Juhuang Song, Fugui Zhang, Lingfei Qi, Hao Cao, Yuan Wang, Zutao Zhang, Jinyue Yan

**Affiliations:** 1 School of Mechanical Engineering, Guizhou University, Guiyang Guizhou 550025, P.R. China; 2 School of Mechanical Engineering, Southwest Jiaotong University, Chengdu 610031, China; 3 Department of Building Environment and Energy Engineering, The Hong Kong Polytechnic University, Hongkong, China; 4 School of Business, Society and Energy, Mälardalen University, 72123 Västerås, Sweden

**Keywords:** Applied sciences, Energy systems

## Abstract

In this paper, three new important aspects of rotary electromagnetic vibration energy harvesting technology (RE-VEH) are concerned and investigated: (i) vibro-electric coupling mechanism of the RE-VEH system is studied through theoretical modeling; (ii) quantitative analysis of system parameters based on numerical simulation method is carried out for the optimal design of RE-VEH; and (iii) dynamic power output performance of the RE-VEH system in free vibration is discussed. The parameter adjusting methods of the RE-VEH system in free vibration mode are obtained through theoretical analysis and numerical simulation. The experimental results show that the power output performance of RE-VEH in free vibration mode matches the numerical simulation results. The simulation and experimental results show that the maximum voltage output and power output of the RE-VEH with different structure parameters under free vibration can be up to the level of 10^0^∼10^1^ V/watt. The above results indicate that RE-VEH in a free vibration environment has significant energy output performance.

## Highlights

• Vibro-electric coupling model of the RE-VEH system under free vibration is established
• Numerical simulation of the vibro-electric coupling model is conducted
• Quantitative analysis of the system structure parameters is carried out
• Parameter optimization method for the RE-VEH is proposed

## Graphical abstract

**Figure:**
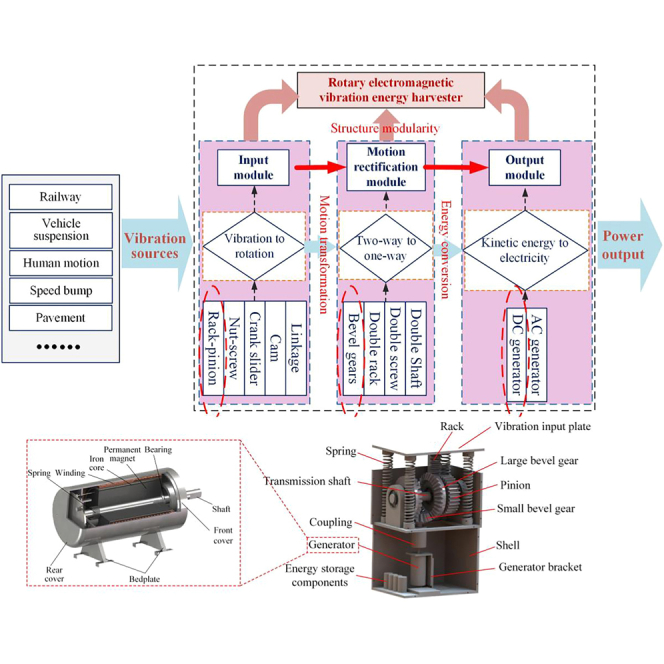

## Introduction

With the advent of the Internet of Things era and the big data age, wide-area distributed wireless network sensor nodes have been widely introduced for environmental state monitoring.^1^^,^^2^ In order to avoid the waste of resources and environmental pollution caused by chemical batteries, clean and decentralized micro-energy technology has attracted great attention of researchers all over the world. Vibration mechanical energy, due to its wide range and various forms, is gradually considered as a kind of clean energy with considerable renewable potential.^3^^,^^4^ Naturally, vibration energy harvesting technology has flourished over the past two decades. In general, vibration energy harvesting technology mainly includes three categories: piezoelectric vibration energy harvesting,^5^^,^^6^^,^^7^ electrostatic vibration energy harvesting,^8^^,^^9^^,^^10^^,^^11^^,^^12^^,^^13^ and electromagnetic vibration energy harvesting.^14^^,^^15^^,^^16^ It is worth noting that electromagnetic vibration energy harvesting technology can be further subdivided into linear electromagnetic vibration energy harvesting technology^17^ and rotary electromagnetic vibration energy harvesting technology (RE-VEH).^18^ Among aforementioned vibration energy harvesting technologies, RE-VEH has become a particularly prominent research hotspot in the field of vibration energy harvesting due to its superior performance in power output.

Principally, the RE-VEH refers to using a set of motion transmission systems to convert the linear vibration into rotation, and then transfer it to the generator for power generation.^19^ Structurally, the rotating electromagnetic vibration energy harvesting system mainly includes three modules: vibration input module, mechanical motion rectification module, and power generation module.^20^^,^^21^^,^^22^ In recent years, RE-VEH based on different mechanical structures emerge in an endless stream. In particular, the update speed of the vibration input module and the mechanical motion rectification module is notably fast.

The function of the vibration input module is to convert linear vibration into rotational motion. Vibration input modules based on different mechanisms have been extensively studied, such as rack and pinion, nut screw, crank slider, cam mechanism, and space linkage.^23^ For example, Zhang et al. proposed rack-pinion-based energy regenerative shock absorber which was used for scavenging vibration energy from vehicle suspension.^24^ Gholikhani et al. separately designed a linear generator-based VEH and a rack-pinion-based RE-VEH to capture vibration energy from speed bump.^25^ The experimental results showed that the root mean square of power output of the vibration energy harvester based on rack and pinion and linear generator was 1.2 W and 80 mW, respectively. Besides, Salman et al. studied a regenerative absorber which used nut-screw mechanism as the vibration input module to harvest vibration energy from vehicle.^26^ The bench test results showed that a peak efficiency of 52% and an average efficiency of 40% could be obtained. Li et al. developed a twin slider-crank mechanisms-based vibration energy harvester for powering monitoring sensors in railway cars.^27^ Experiment results showed that the peak phase power was 24.6 W and the average power was 4.8 W under harmonic excitation with an amplitude of 12.5 mm and a frequency of 3 Hz. Kamali et al. proposed an electromagnetic dampers based on cylindrical cam mechanism for harvesting the vibration energy from automotive and bicycle.^28^ In addition, Maravandi and Moallem studied a space 2-bar linkage-based regenerative shock absorber, and the experiment results showed that mechanical energy conversion efficiency of the proposed regenerative shock absorber was considerably higher than that of other mechanisms-based regenerative shock absorber.^29^

As for the mechanical motion rectification module, its function is to convert bidirectional rotation into unidirectional rotation. There are mainly four mechanisms that are considered as mechanical motion rectification modules, including double rack-pinion, bevel gear set, double screw, and double gear train. Liu et al. proposed a vibration energy harvesting system based on double rack-pinion.^30^ Simulation was carried out under harmonic excitations of constant displacement amplitude, and the results showed that the bandwidth of the motion rectification mechanism-based vibration energy harvester was broader than that of the counterpart without motion rectification. Ali et al. proposed a regenerative shock absorber using a set of bevel gears as motion rectification to convert bidirectional rotation into one-way rotation.^31^ The experiment results showed that the peak output power of the proposed regenerative shock absorber could be up to 3.85 W at 7.5 mm sinusoidal amplitude and 2 Hz input frequency. Wang et al. designed a double screw as the motion rectification mechanism for the regenerative shock absorber.^32^ The results showed that an average power output of 3.701 W in 1 Hz-3 mm sinusoidal vibration input could be obtained. In addition, Zhang et al. developed a railway vibration energy harvesting system based on double gear transmission system.^33^ The simulation results demonstrated that the proposed energy harvester had a rapid response.

Although the rotary electromagnetic vibration energy harvesting technology has become a global research hotspot, there are still some problems in previous studies: (ⅰ) almost only the structure and application of the vibration energy harvesting system have been focused on and discussed, resulting in the energy harvesting system not having the optimal parameter configuration; (ⅱ) only sinusoidal input excitation has been considered as the input of the vibration energy harvesting system, resulting in the energy harvesting system not being suitable for other forms of excitation; and (iii) how to optimize the output performance of RE-VEH has not been studied. In this paper, three new aspects of RE-VEH are focused and studied: (a) vibro-electric coupling mechanism of the RE-VEH based on classic vibration input module and motion rectification module is studied through theoretical modeling; (b) the system structure parameter adjusting method for optimal design of RE-VEH is investigated; and (c) the dynamic power output characteristics of the RE-VEH in free vibration is discussed.

## Results

### Architecture of rotary electromagnetic vibration energy harvesting system

**Figure 1:**
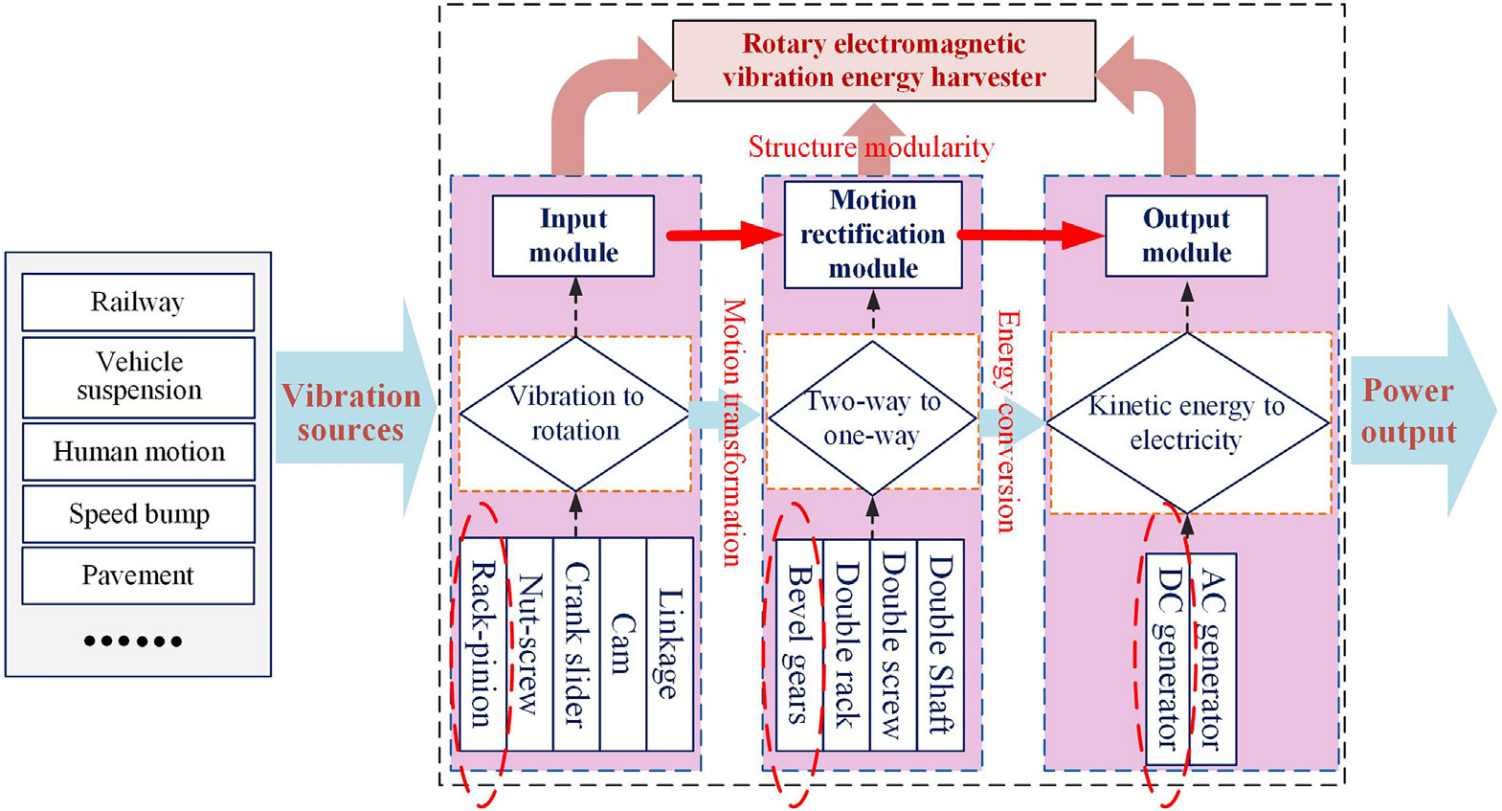
Architecture of rotary electromagnetic vibration energy harvesting technology

In the introduction section, it has been clarified that the RE-VEH includes three elements: vibration input, motion rectification, and power output. The vibration input refers to the excitation caused by vibration from ambient environment, such as railway system, vehicle suspension, speed bump, human motion, etc.; power output refers to the regenerative electricity that can be used for powering the electrical load; motion rectification is the medium that can connect vibration input and power output. Among them, motion rectification is the most important element, which can help RE-VEH system obtain high-quality power output. Correspondingly, the RE-VEH system mainly includes ternary modules in structure, which are vibration input submodule, motion rectification submodule, and power output submodule, as shown in Figure 1.

### Vibration input module

**Figure 2:**
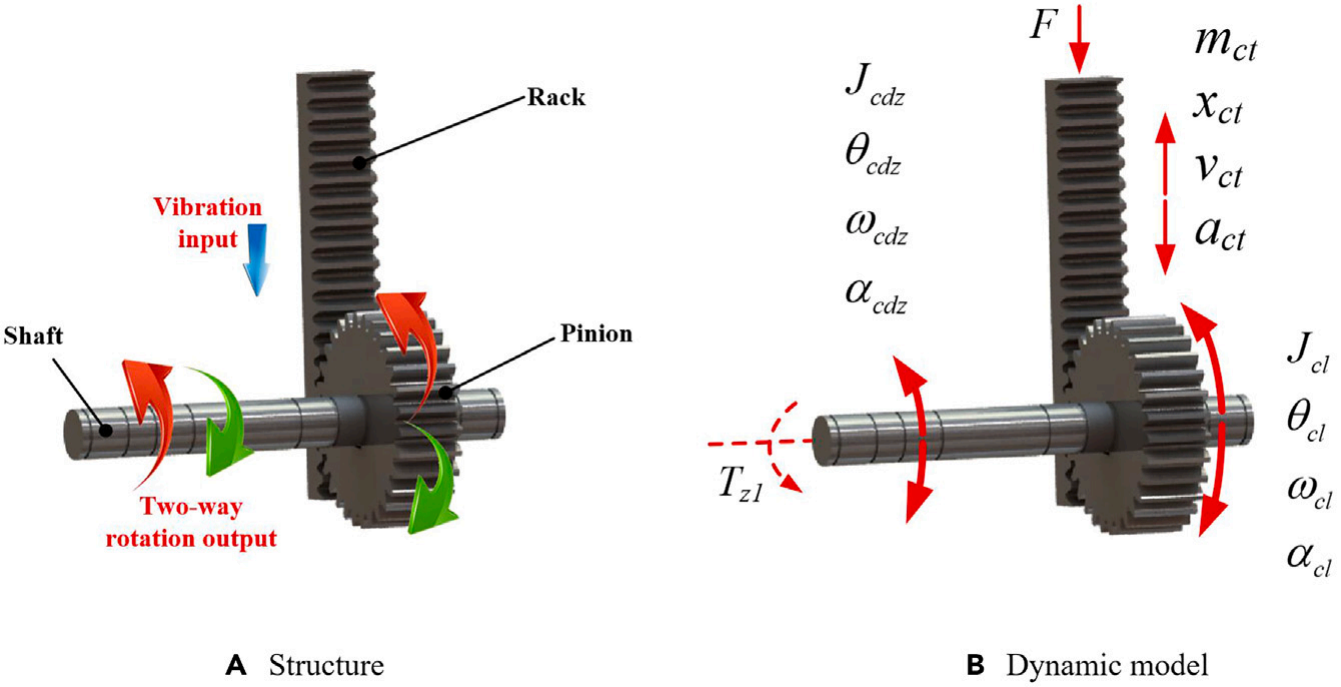
Vibration input module (A) Structure. (B) Dynamic model.

The structure of the vibration input module is shown in Figure 2A, including a rack, a gear, and a transmission shaft. The motion transmission process is as follows. The external vibration drives the rack to reciprocate linearly and then drives the gear meshing with the rack to rotate back and forth. Next, the gear transmits the two-way rotation to the shaft which is fixed with the gear. Finally, the shaft outputs the bidirectional rotation to next module. Based on the 3D model design, the dynamic model of the vibration input module is shown in Figure 2B. In this figure, *F* is the external excitation force; *m*_*ct*_, *x*_*ct*_, *v*_*ct*_, and *a*_*ct*_ are the mass, linear displacement, linear velocity, and linear acceleration of the rack, respectively; $J_{cl}$, $\theta_{cl}$, $\omega_{cl}$, and $\alpha_{cl}$ are the moment of inertia, angular displacement, angular velocity, and acceleration of the gear, respectively; $J_{cdz}$, $\theta_{cdz}$, $\omega_{cdz}$, and $\alpha_{cdz}$ are the moment of inertia, angular displacement, angular velocity, and angular acceleration of the transmission shaft, respectively; and *T*_*z1*_ is the resistance torque acting on the transmission shaft by the next module.

### Mechanical motion rectification module

**Figure 3:**
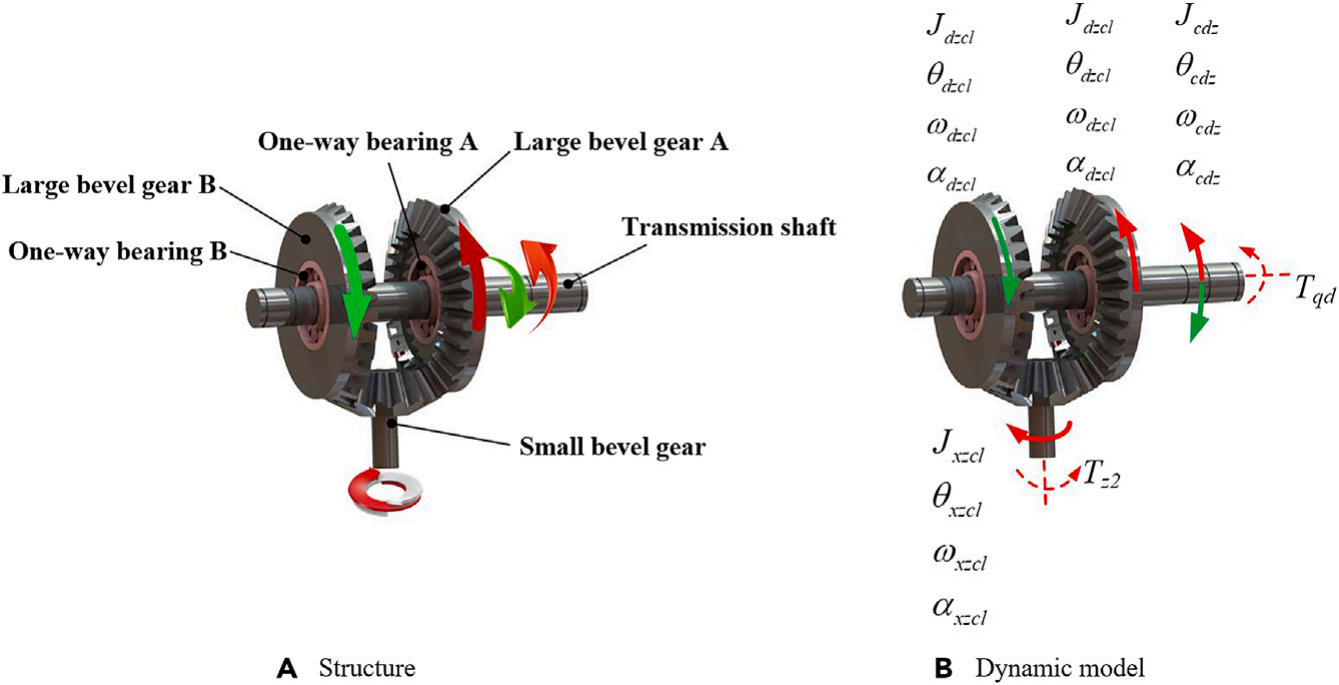
Motion rectification module (A) Structure. (B) Dynamic model.

When the vibration is transmitted to the input module, its motion form will be transformed into a rotational motion with alternating directions, and this motion will cause the rotation direction of the input shaft of the generator to change alternately. In order to further improve the efficiency and stability of energy output, it is necessary to convert the bidirectional rotational motion into unidirectional rotation and then transmit it to the energy output module. In this paper, the motion rectification mechanism based on bevel gear set is considered as the motion transmission module of the energy capture system, of which the structure and motion transmission process are shown in Figure 3A.

Based on the 3D model design, the dynamic model of the motion rectification module is shown in Figure 3B. In this figure, *T*_*qd*_ is the torque applied by the vibration input module on the transmission shaft, and its magnitude is equal to *T*_*cdz*_. $J_{cdz}$, $\theta_{cdz}$, $\omega_{cdz}$, and $\alpha_{cdz}$ are the moment of inertia, angular displacement, angular velocity, and angular acceleration of the transmission shaft, respectively; $J_{dzcl}$, $\theta_{dzcl}$, $\omega_{dzcl}$, and $\alpha_{dzcl}$ are the moment of inertia, angular displacement, angular velocity, and angular acceleration of the large bevel gear and the one-way bearing combination, respectively; $J_{xzcl}$, $\theta_{xzcl}$, $\omega_{xzcl}$, and $\alpha_{xzcl}$ are the moment of inertia, angular displacement, angular velocity, and angular acceleration of the small bevel gear, respectively. *T*_*z2*_ is the torque acting on the small bevel gear by the subsequent energy output module.

### Output module

**Figure 4:**
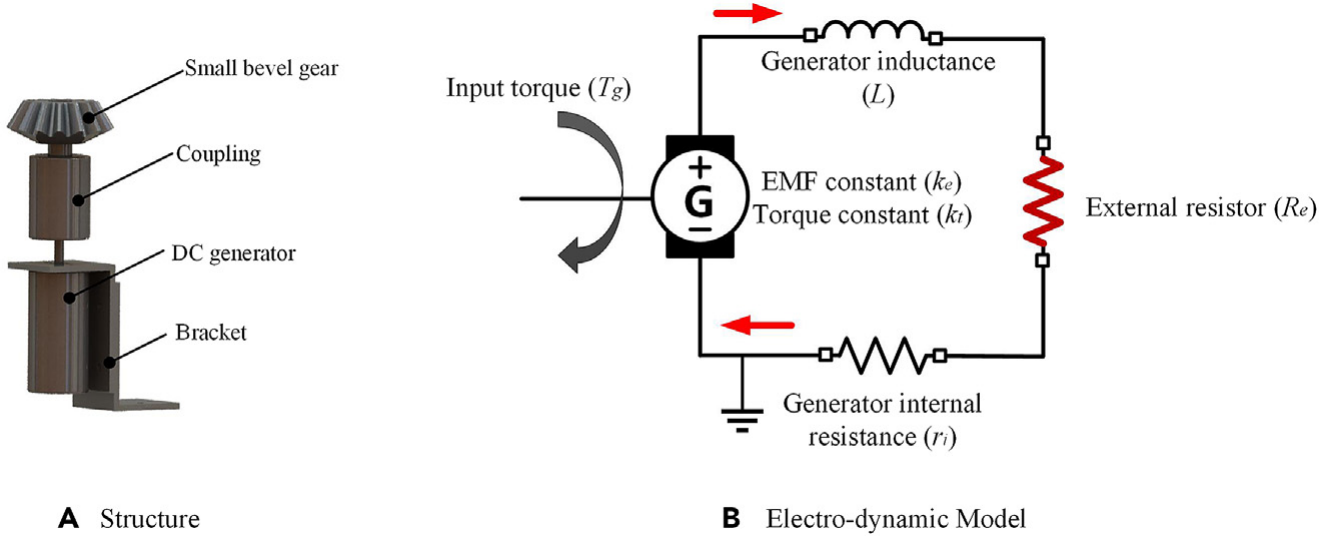
Power output module (A) Structure. (B) Electro-dynamic model.

The output module of the vibration energy harvesting system is generator. In general, the types of generator include alternating current (AC) generator and direct current (DC) generator, both of which have their own advantages and disadvantages. The alternator has high power generation efficiency, but the output electricity is an AC that varies in magnitude and direction. Although the power generation efficiency of the DC generator is not as good as that of the AC generator, its output electricity is pulsating DC, which is closer to the power demand of the micro power load. In this paper, the DC generator is used as the power output module of the vibration energy harvesting system to analyze its electrodynamic mechanism. The structure of the output module is shown in Figure 4A. The input shaft of the DC generator is connected to the small bevel gear through a coupling, and the generator body is fixed to the generator bracket. The electrodynamic model of the output module is shown in Figure 4B. When the external excitation drives the input shaft of the generator to rotate, the electromagnetic coil of the generator will generate an induced current *i*, which will generate a resistance torque *T*_*i*_ applied to the input shaft of the generator.

### Vibro-electric coupling model

**Figure 5:**
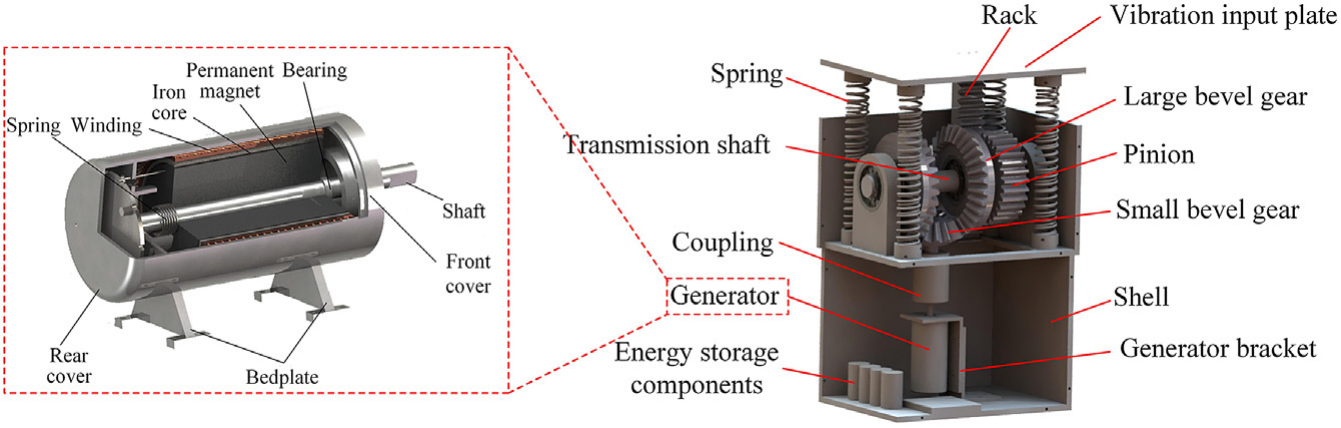
General structure of RE-VEH based on rack-pinion and bevel gears mechanism

**Figure 6:**
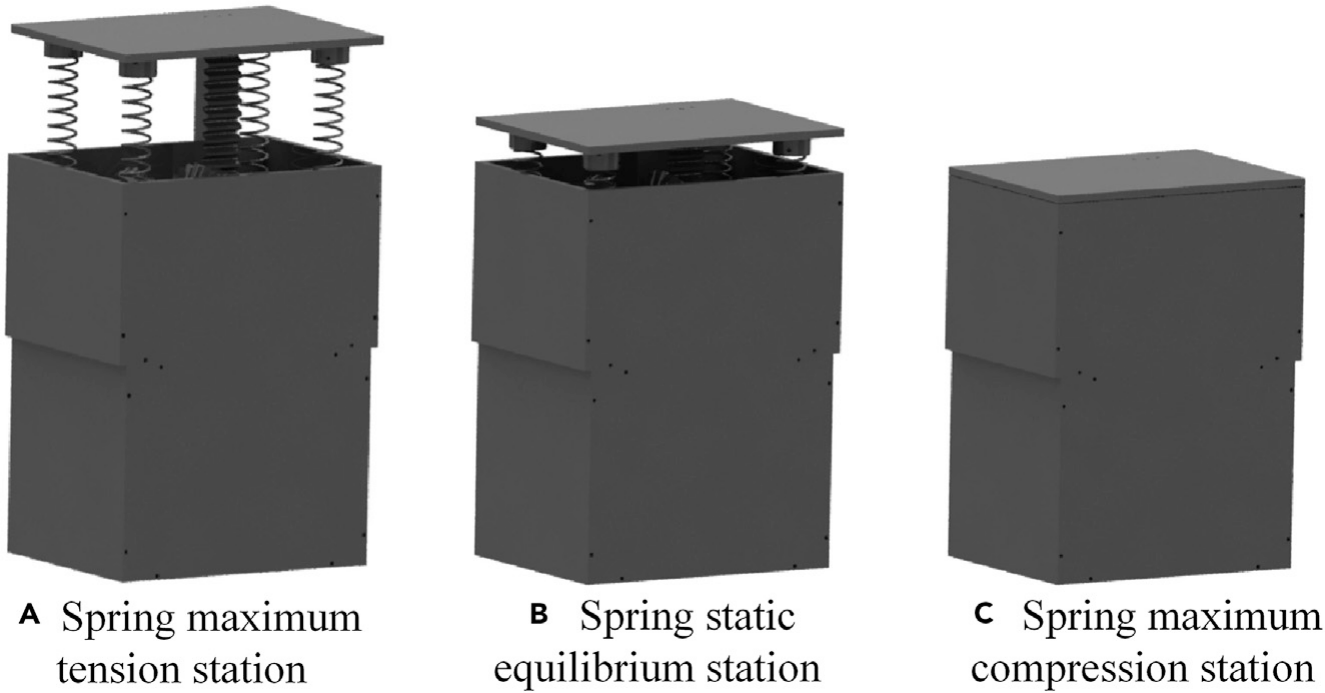
Three different stations of the VEH system (A) Spring maximum tension station. (B) Spring static equilibrium station. (C) Spring maximum compression station.

**Figure 7:**
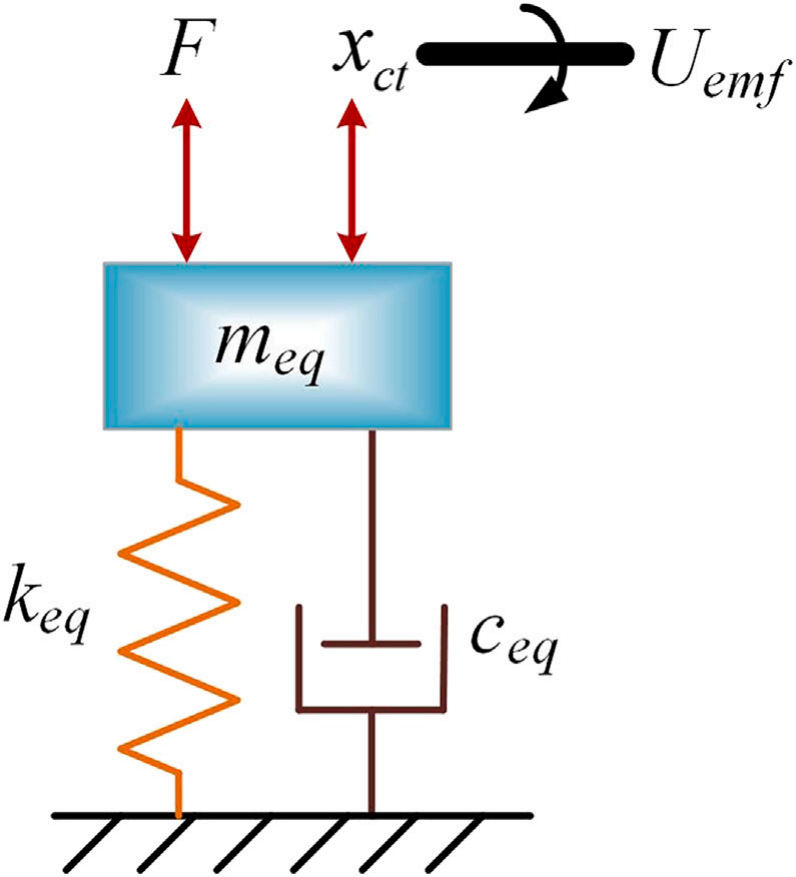
The equivalent vibro-electric coupling model of the RE-VEH

The overall structure of the RE-VEH system with the rack-pinion as the input module, the bevel gear set as the motion rectification module, and the DC generator as the output module is shown in Figure 5. The components of the system include rack, vibration input plate, large bevel gear, pinion, small bevel gear, spring, transmission shaft, coupling, generator, shell, generator bracket, and energy storage components. Figure 6 shows the three different stations of the vibration energy harvesting system, including the tension station, the static equilibrium station, and the compression station. It can be seen from this figure that there is a gap between the vibration input plate and the protective shell in the static equilibrium station. Therefore, when the RE-VEH is subjected to an external force, it can be compressed. The equivalent vibro-electric coupling model of the RE-VEH is depicted in Figure 7.

## Discussion

### Influence of equivalent mass parameters on power output performance

**Figure 8:**
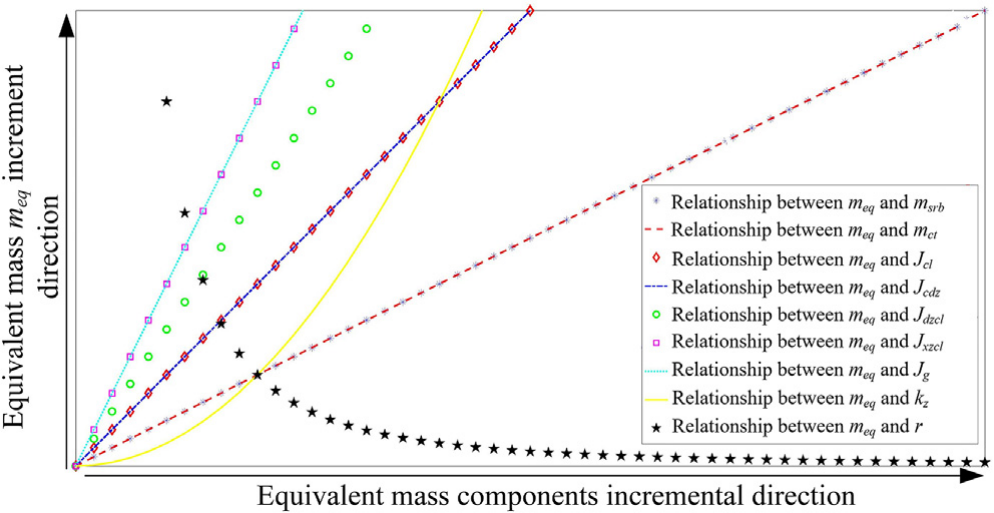
The relationship between the equivalent mass of RE-VEH and each mass component

The equivalent mass of the system is composed of the vibration input plate mass, rack mass, shaft rotational inertia, gear rotational inertia, large and small bevel gear rotational inertia, generator rotational inertia, bevel gear ratio, and opinion diameter. It is particularly important to study the relationship between the various components of system equivalent mass and the power output for the optimal design of the RE-VEH system. Figure 8 shows the relationship between the equivalent mass of the system and its component. It can be seen from this figure that the vibration input plate mass and rack mass have the same linear positive correlation with the system equivalent mass; the rotational inertia of the pinion and transmission shaft have the same linear positive correlation with the system equivalent mass; the rotational inertia of the small bevel gear and the generator also have the same linear positive correlation with the system equivalent mass; the rotational inertia of the large bevel gear has a linear positive correlation with the system equivalent mass; the equivalent mass of the system presents a parabolic upward trend with the increase of the transmission ratio between the large and small bevel gears; the equivalent mass of the system decreases with the increase of the pinion radius, but its rate of change decreases gradually with the increase of the pinion radius. Although the relationship between the mass components and the system equivalent mass has been clarified, some of the mass elements are also involved in the constitution of equivalent damping. Therefore, it is necessary to discuss the direct influence of each element of equivalent mass on the power output performance of the RE-VEH for further enhancing the system performance.

**Figure 9:**
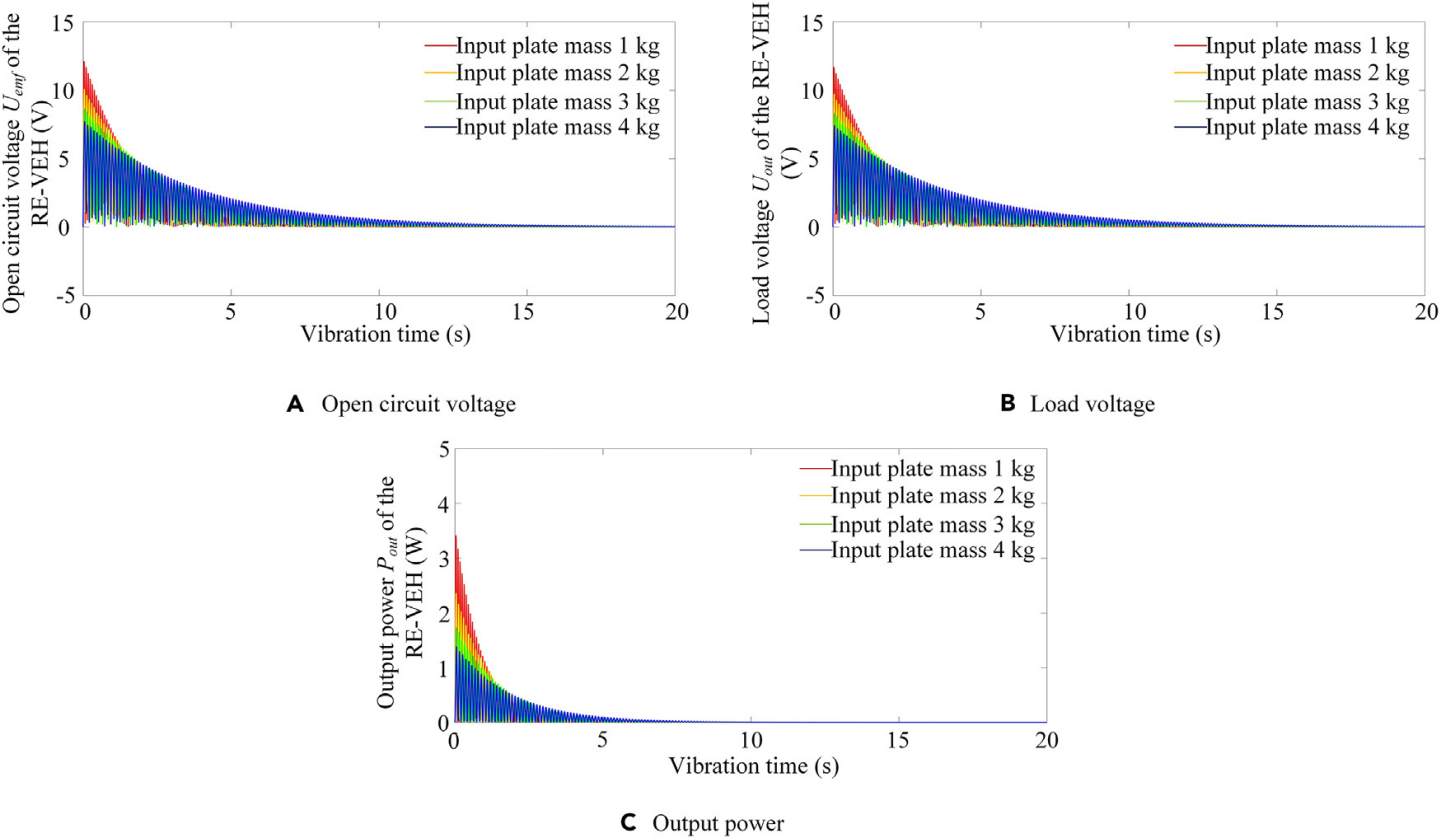
Dynamic power output of RE-VEH under different input plate mass (A) Open circuit voltage. (B) Load voltage. (C) Output power.

Since the vibration input plate mass and rack mass have the same linear positive correlation with the system equivalent mass, therefore, this paper only discusses the system power output under different vibration input plate mass. Figure 9 shows the variation trends of the open-circuit output voltage (*U*_*emf*_), the load output voltage (*U*_*out*_), and the output power *P*_*out*_ of the RE-VEH under different vibration input plate mass (*m*_*srb*_). It can be seen from this figure that the power output of the vibration energy harvesting system decays to 0 with time under free vibration mode, which is in line with the dynamic response characteristics of free vibration system. Secondly, the open-circuit voltage and load voltage are always positive over time, which proves that the energy harvester itself can achieve rectification. Thirdly, it can be seen from Figures 9A, (B), and (C) that when the *m*_*srb*_ is larger, the open-circuit voltage, load voltage, and output power decay more slowly, and their peak values (at the start of vibration) are smaller. In general, the peak power output and output smoothness of the RE-VEH can be improved by adjusting the mass of the vibration input plate.

**Figure 10:**
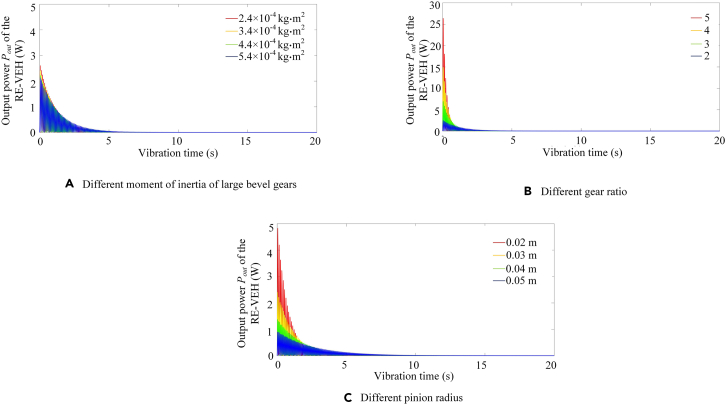
Dynamic output power of RE-VEH under different mass parameters (A) Different moment of inertia of large bevel gears. (B) Different gear ratio. (C) Different pinion radius.

Figure 10 shows the trend of dynamic power output of the RE-VEH when the other three mass component parameters change. From Figure 10A, it can be seen that the smaller the moment of inertia of the large bevel gear is, the greater the peak power output of the system can be obtained. In general, the change of the moment of inertia has little effect on the power output of the system. From Figure 10B, it can be obtained that with the increase of the transmission ratio, the output power of the system will increase significantly. Besides, it can be seen from Figure 10C that as the pinion radius decreases, the output power of the system also decreases. By comparing Figures 10A, (B), (C), and Figure 9C, it can be concluded that the output power of the system is most affected by the system transmission ratio, followed by the pinion radius, the mass of the moving parts, and the moment of inertia of the rotating parts.

### Influence of equivalent damping parameters on power output performance

**Figure 11:**
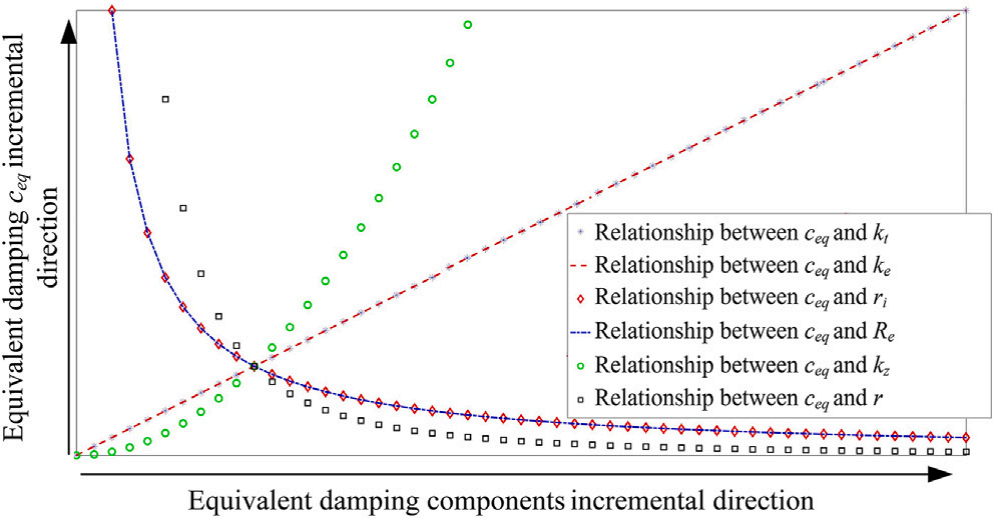
The relationship between the equivalent damping and each component of the equivalent damping

According to the theoretical analysis, the equivalent damping coefficient of the system is composed of the generator back electromotive force (EMF) constant (*k*_*e*_), the generator torque constant (*k*_*t*_), the generator internal resistance (*r*_*i*_), the system transmission ratio (*k*_*z*_), the pinion radius (*r*), and the external load resistance (*R*_*e*_). First, the approximate relationship between the equivalent damping coefficient of the system and its various components can be solved, as shown in Figure 11. It can be seen from this figure that the torque constant and back EMF constant of the generator have the same linear positive correlation with the equivalent damping coefficient of the system. The internal resistance of the generator and external load resistance have the same nonlinearity negative correlation with the equivalent damping coefficient. With the increase of generator internal resistance and external load resistance, the change rate of equivalent damping coefficient will become smaller. There is a nonlinear positive correlation between system transmission ratio and system equivalent damping coefficient. As the transmission ratio increases, the change rate of the equivalent damping coefficient will increase. There is a nonlinear negative correlation between the pinion radius and the system equivalent damping coefficient. As the pinion radius increases, the change rate of the equivalent damping coefficient decreases. The effects of each component of the equivalent damping coefficient on the power output of the RE-VEH are discussed separately in the following.

**Figure 12:**
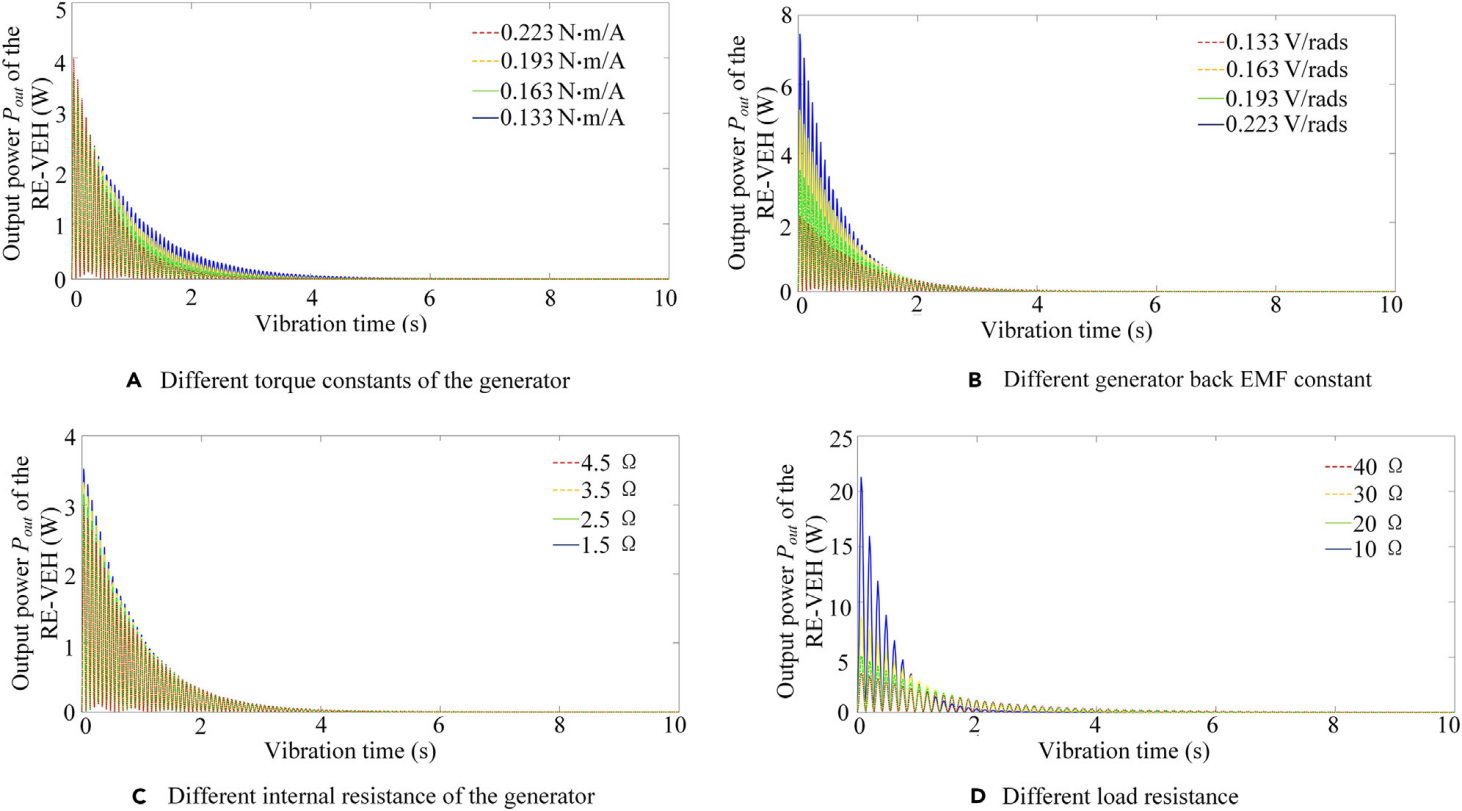
Dynamic output power of RE-VEH under different damping parameters (A) Different generator torque constants. (B) Different generator back EMF constant. (C) Different generator internal resistance. (D) Different load resistance.

Since the influence of pinion radius and system transmission ratio on power output performance has been discussed in previous section, this section only analyzes the influence of generator torque constant, generator back EMF constant, generator internal resistance, and load resistance on the dynamic power output of the RE-VEH. Figure 12 shows the dynamic output power of the RE-VEH under different generator torque constants, different generator back EMF constants, and different generator internal resistance and load resistance. It can be seen from Figure 12A that, as the torque constant of the generator increases, the initial value of the output power increases slightly, and the decay rate of system output power also has a slight increasing trend. It can be seen from Figure 12B that with the increase of the generator back EMF constant, the system power output can be efficiently improved. It can be seen from Figure 12C that generator internal resistance has little effect on system power output. From Figure 12D, we can obtain that when the load resistance is close to the internal resistance of the generator (*r*_*i*_ = 1.5 Ω), the system output power can be significantly improved.

### Influence of equivalent stiffness parameters on power output performance

**Figure 13:**
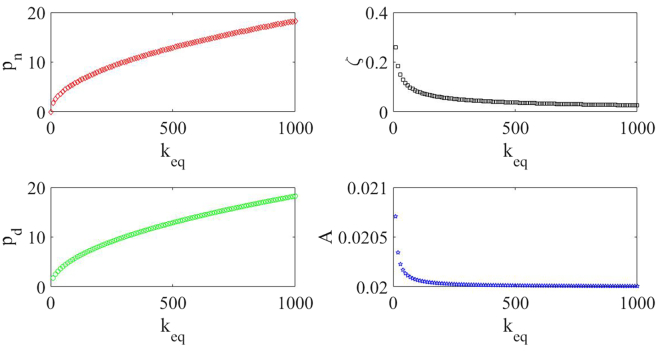
The relationship between the equivalent stiffness and four dynamic characteristic parameters of the RE-VEH

**Figure 14:**
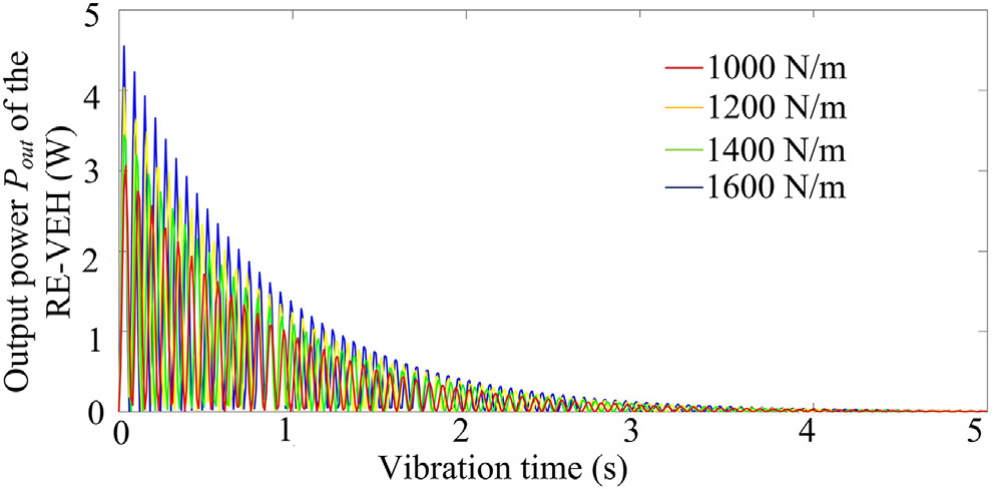
Dynamic output power of the RE-VEH under different spring stiffness coefficients

In addition to equivalent mass and equivalent damping, equivalent stiffness is also a key parameter that affects the dynamic response characteristics of the vibration energy capture system. Figure 13 shows the relationship between the equivalent stiffness (*k*_*eq*_) and four dynamic characteristic parameters of the RE-VEH. It can be seen from the figure that with the increase of the equivalent stiffness, both the undamped natural circular frequency (*p*_*n*_) and the damped natural circular frequency (*p*_*d*_) of the system show an upward trend, while the damping ratio (*ζ*) and the maximum vibration amplitude (*A*) of the system gradually decrease to saturation state. The selection of spring can be directly determined by the relationship between the spring stiffness and the dynamic output power of the RE-VEH. Figure 14 shows the dynamic output power of the RE-VEH under different spring stiffness coefficients. It can be seen from this figure that that the greater the spring stiffness coefficient, the higher the dynamic power output of the system.

### Experiment test

**Figure 15:**
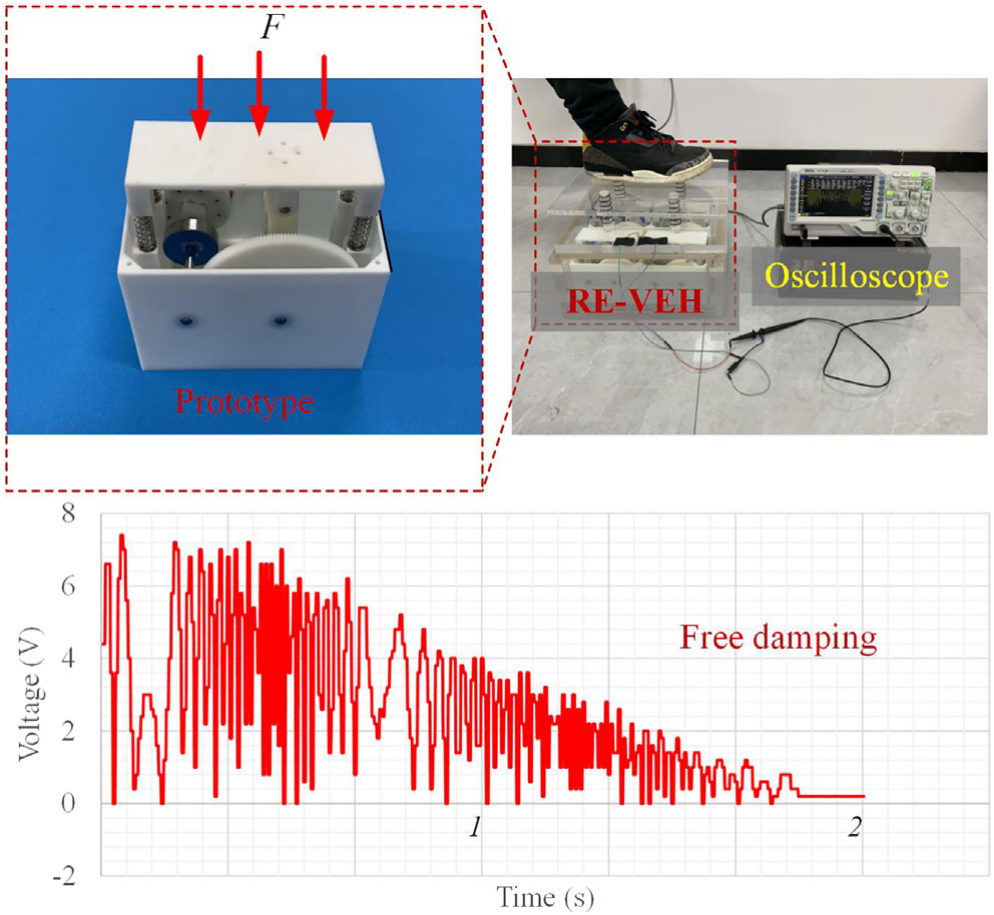
Experiment test and results

In order to verify the theoretical model and simulation analysis results, the prototype of the RE-VEH has been fabricated to conduct experiment test as shown in Figure 15. The oscilloscope is used to display and store the voltage signal output of the energy harvesting system. Based on the simulation analysis, the optimal parameter configuration is selected for manufacturing the RE-VEH system. In order to enhance the waterproof performance, the materials of input board, gasket, and case are all acrylic sheets. Four cylinders attached on the input board were used to transfer the force of human body when walker stepped on the lid as shown in Figure 15. According to dynamics and electrical analysis, the damped free vibration of the input board is mainly affected by force excitation, which affects the operating speed of the internal system and thus the power output of the generator. The normal walking speed of an average adult is about 1.1 m/s to 1.5 m/s; hence the walking speed was set 1.2 m/s in this manuscript. Under normal walking conditions, the magnitude of the excitation force applied to the input board is almost solely related to the weight of the human body. The speed and acceleration of human walking only affect the response speed of the system and have almost no impact on the power output of the system. We choose the weight of human ranged from 50 kg to 80 kg to step on the lid. This paper uses human stepping to simulate the instantaneous excitation force on the energy harvesting system. After stepping, the motion displacement of the energy harvesting system will gradually decay under the synergistic effect of springs and electromagnetic damping. As shown in Figure 15, the power generation trend of the energy harvesting system also shows a gradual attenuation trend, which is consistent with the numerical simulation results.

**Figure 16:**
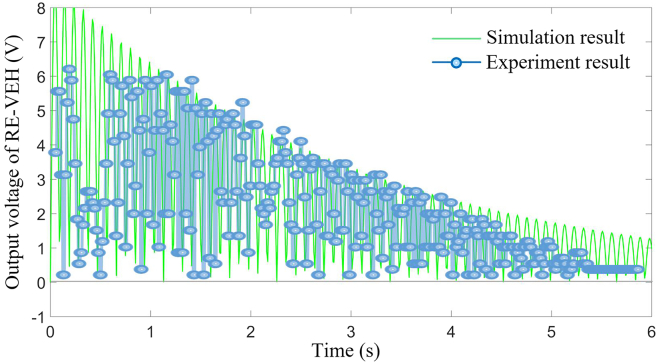
Comparison between experimental and simulation results

As shown in Figure 16, both simulation and experimental results demonstrate that under instantaneous excitation, the power output of the system will gradually decay as the system undergoes damped free vibration, as shown in following figure. Therefore, in terms of overall trend, the simulation and experimental results are consistent, indicating that the theoretical analysis is basically in line with the actual situation. However, due to errors in machining accuracy and installation of the actual RE-VEH system, there is also a certain degree of error between simulation data and experimental data.

**Figure 17:**
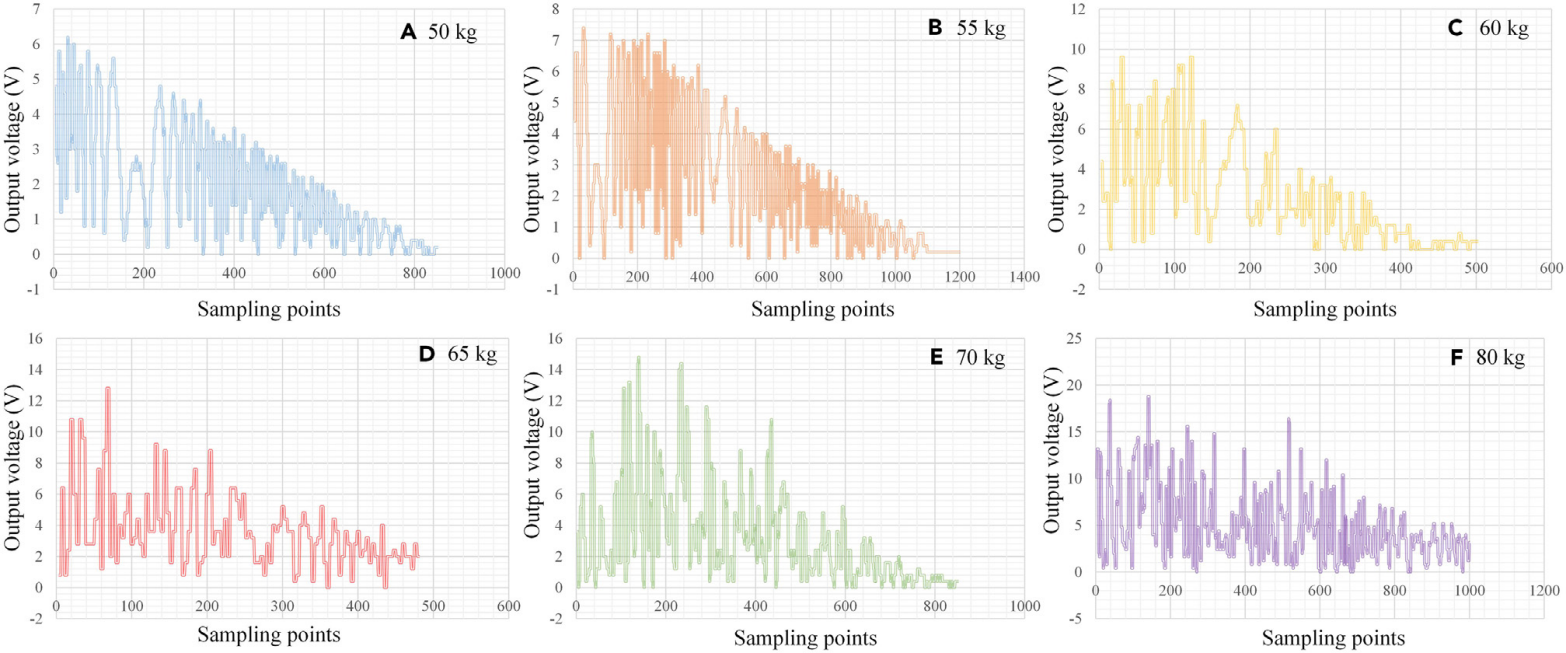
Experimental output voltage of the VEH under different human body weights A. 50 kg. B. 55 kg. C. 60 kg. D. 65 kg. E. 70 kg. F. 80 kg.

Figure 17 shows the output voltage of the vibration energy harvesting system under human stepping with different weights. From this figure, it can be seen that as the weight of the human body increases, the maximum voltage output of VEH will also increase. Even under the excitation of only 50 kg weight, the output voltage of this VEH is not less than 6 V. When the excitation weight reaches 80 kg, the maximum output voltage of VEH can be up to 15 V, which is consistent with simulation results. In addition, the short-circuit current changes from 19.1 mA to 39.2 mA. Therefore, the output power can be calculated by multiplying the voltage and current values, which range from 114.6 mW to 588 mW. The weight and volume of the VEH prototype are 3 kg and 0.018 m^3^, respectively. Therefore, the mass power density and volume power density of the proposed VEH can be obtained, which can be up to 196 mW/kg and 32.7 W/m^3^, respectively. Based on the aforementioned results, it can be concluded that the power output performance of the energy harvesting system is very impressive in the free vibration mode, indicating that deploying electromagnetic vibration energy harvesting systems in free vibration environments such as road surfaces and speed bumps has significant energy benefits.

### Conclusions

In this paper, the dynamic power output and optimization parameter adjustment method of the RE-VEH in free vibration mode are studied. Firstly, the structure of the RE-VEH is summarized according to the previous research. Secondly, based on the typical module combination (rack and pinion-bevel gears-DC generator), the structure and dynamics of each submodule of RE-VEH are modeled and analyzed. The vibration-electric coupling model of the entire RE-VEH system is established through integrating each submodule. Based on the vibration-electric coupling model, the dynamic response model of the power output of the RE-VEH under free vibration is investigated. The system parameters of the power output model are quantitatively analyzed using numerical simulation method, and the effects of the system equivalent mass, equivalent damping, and equivalent stiffness on the power output performance of the system are studied. The numerical simulation results show that increasing the system transmission ratio *k*_*z*_, making the load resistance *R*_*e*_ close to the generator internal resistance *r*_*i*_, reducing the gear radius *r*, increasing the generator back EMF constant *k*_*e*_, reducing the mass of each moving part (*m*_*srb*_*/m*_*ct*_), and increasing the spring stiffness coefficient *k*_*s*_ can effectively improve the power output performance of the system, while the generator torque constant *k*_*t*_, the moment of inertia of the rotating components (*J*_*dzcl*_*/J*_*cl*_*/J*_*xzcl*_*/J*_*cdz*_*/J*_*g*_), and the generator internal resistance *r*_*i*_ have little effect on the system power output. The experimental results show that the power output performance of RE-VEH in free vibration mode matches the numerical simulation results. The simulation and experimental results show that the maximum voltage output and power output of the RE-VEH with different structure parameters under free vibration can be up to the level of 10^0^–10^1^ V/watt. The short-circuit current changes from 19.1 mA to 39.2 mA. By multiplying the voltage and current values, the output power of the VEH ranges from 114.6 mW to 588 mW. Besides, the mass power density and volume power density can be up to 196 mW/kg and 32.7 W/m^3^, respectively. The aforementioned results lay the foundation for the optimal design and performance enhancement of RE-VEH in the future.

### Limitations of the study

This study only investigates vibration energy harvesting performance and parameter optimization analysis of the proposed energy harvester in free vibration mode. In practical scenarios, there are also forced vibration forms. In the future, it is necessary to study the performance of vibration energy harvesting in forced vibration mode.

## STAR★Methods

### Key resources table

**Table:** 

REAGENT or RESOURCE	SOURCE	IDENTIFIER
**Software and algorithms**		

Matlab 6.0	Matlab	http://b.zhr33.cn/matlab/
Microsoft Visio 2016	Microsoft Visio	https://www.microsoft.com/zh-cn/microsoft-365/visio/flowchart-software
Microsoft Excel 2013	Microsoft Excel	https://www.microsoft.com/zh-cn/microsoft-365/excel

### Resource availability

#### Lead contact

Further information and requests for resources should be directed to and will be fulfilled by the Lead Contact, Lingfei Qi (lfqi@gzu.edu.cn).

#### Materials availability

Not applicable.

### Method details

#### Dynamic model of vibration input module

The relationship between the linear displacement, linear velocity and acceleration of the rack can be expressed as

(Equation 1) $$\left\{ \begin{array}{l} v_{ct}=\frac{dx_{ct}}{dt}\\ a_{ct}=\frac{dv_{ct}}{dt}=\frac{d^{2}x_{ct}}{dt^{2}} \end{array}\right.$$

In the same way, the relationship between the angular displacement, angular velocity and angular acceleration of the gear and transmission shaft can be expressed as

(Equation 2) $$\left\{ \begin{array}{l} \omega_{cl}=\frac{d\theta_{cl}}{dt}\\ \alpha_{cl}=\frac{d\omega_{cl}}{dt}=\frac{d^{2}\theta_{cl}}{dt^{2}} \end{array}\right.$$

(Equation 3) $$\left\{ \begin{array}{l} \omega_{cdz}=\frac{d\theta_{cdz}}{dt}\\ \alpha_{cdz}=\frac{d\omega_{cdz}}{dt}=\frac{d^{2}\theta_{cdz}}{dt^{2}} \end{array}\right.$$

According to D'Alembert's principle, the dynamics of the rack can be expressed as

(Equation 4) $$F=m_{ct}\cdot a_{ct}+F_{cl}$$

where *F*_*cl*_ is the force acting on the rack by the gear. Ignoring the jamming phenomenon and friction between the rack and pinion, the dynamics of the gear is

(Equation 5) $$F_{cl}\cdot r=J_{cl}\cdot \alpha_{cl}+T_{cdz}$$

where *r* is the indexing circle radius of the gear, and *T*_*cdz*_ is the torque acting on the gear by the transmission shaft. Taking the transmission shaft as the object, its dynamics can be expressed as

(Equation 6) $$T_{cdz}=J_{cdz}\cdot \alpha_{cdz}+T_{z1}$$

Besides, the relationship between the angular velocity of the gear and the linear velocity of the rack can be expressed as

(Equation 7) $$v_{ct}=\omega_{cl}\cdot r$$

Since the gear and the transmission shaft are fixedly matched, the relationship between their angular displacement, angular velocity and angular acceleration can be described as

(Equation 8) $$\theta_{cl}=\theta_{cdz};\;\omega_{cl}=\omega_{cdz};\;\alpha_{cl}=\alpha_{cdz}$$

Therefore, by combining Equation 1 to Equation 8, the dynamic model of the vibration input module can be obtained by

(Equation 9) $$F=\left(m_{ct}+\frac{J_{cl}+J_{cdz}}{r^{2}}\right)\cdot \frac{d^{2}x_{ct}}{dt^{2}}+\frac{T_{z1}}{r}$$

#### Dynamic model of mechanical motion rectification module

First, when the transmission shaft rotates in the direction of the red arrow, its dynamic equation is

(Equation 10) $$T_{qd}=J_{cdz}\cdot \alpha_{cdz}+T_{dzcl-A}$$

where *T*_*dzcl-A*_ is the resistance torque acting on the transmission shaft by the large bevel gear A, which is equivalent to *T*_*z1*_. Ignoring the tooth jamming and friction between the bevel gears, taking the large bevel gear A as the research object, its dynamics can be expressed as

(Equation 11) $$T_{dzcl-A}=J_{dzcl}\cdot \alpha_{dzcl}+k_{z}\cdot T_{xzcl}$$

where *k*_*z*_ is the transmission ratio between the large bevel gear and the small bevel gear, and *T*_*xzcl*_ is the driving torque obtained by the small bevel gear. Taking the bevel gear as the object, its dynamics is

(Equation 12) $$T_{xzcl}=J_{xzcl}\cdot \alpha_{xzcl}+T_{z2}+\frac{T_{dzcl-B}}{k_{z}}$$

where *T*_*dzcl-B*_ is the driving torque obtained by the large bevel gear B. At this time, the large bevel gear B is free to rotate, and its dynamics is

(Equation 13) $$T_{dzcl-B}=J_{dzcl}\cdot \alpha_{dzcl}$$

Therefore, by combining Equation 10 to Equation 13, the dynamics of the entire motion rectifier module can be obtained as

(Equation 14) $$T_{qd}=J_{cdz}\cdot \alpha_{cdz}+J_{dzcl}\cdot \alpha_{dzcl}+k_{z}\cdot \left(J_{xzcl}\cdot \alpha_{xzcl}+T_{z2}+\frac{J_{dzcl}\cdot \alpha_{dzcl}}{k_{z}}\right)=\left(J_{cdz}+2\cdot J_{dzcl}+{k_{z}}^{2}\cdot J_{xzcl}\right)\cdot \frac{d\omega_{cdz}}{dt}+k_{z}\cdot T_{z2}$$

After simplifying the Equation 14, the dynamics of the motion rectifier module can be expressed as

(Equation 15) $$T_{qd}=\left(J_{cdz}+2\cdot J_{dzcl}+{k_{z}}^{2}\cdot J_{xzcl}\right)\cdot \frac{d^{2}x_{ct}}{r\cdot dt^{2}}+k_{z}\cdot T_{z2}$$

#### Electrodynamic model of the output module

The relationship between induced current and resistance torque can be expressed as^34^

(Equation 16) $$T_{i}=k_{t}\cdot i$$

where *k*_*t*_ is the torque constant of the generator. According to Newton's second law, the dynamics of the generator input shaft is

(Equation 17) $$T_{g}-T_{i}=J_{g}\cdot \frac{d^{2}\theta_{g}}{dt^{2}}$$

where *T*_*g*_, *J*_*g*_ and $\theta_{g}$ are the input torque, moment of inertia and rotation angle of the generator, respectively. When the generator produces the induced current, it also generates the induced electromotive force *U*_*emf*_ which can be expressed as

(Equation 18) $$U_{emf}=k_{e}\cdot \omega_{g}=k_{e}\cdot \frac{d\theta_{g}}{dt}$$

where $\theta_{g}$ is the rotation angle of the generator shaft. According to Kirchhoff's voltage law, the following relation can be obtained:

(Equation 19) $$U_{emf}-L\frac{di}{dt}-iR=0$$

where *R* is the sum of the generator internal resistance and external resistance, that is, *R*=*r*_*i*_+*R*_*e*_. Combining Equation 16 to Equation 19, the electrodynamic expression of the generator can be obtained as

(Equation 20) $$k_{e}\frac{d\theta_{g}}{dt}=L\frac{d\left(T_{i}/k_{t}\right)}{dt}+\frac{T_{i}}{k_{t}}R=\frac{L}{k_{t}}\frac{d}{dt}\left(T_{g}-J_{g}\frac{d^{2}\theta_{g}}{dt^{2}}\right)+\frac{R}{k_{t}}\left(T_{g}-J_{g}\frac{d^{2}\theta_{g}}{dt^{2}}\right)$$

By applying Laplace transform to Equation 20, the above equation can be expressed as

(Equation 21) $$T_{g}\left(s\right)=\frac{k_{t}\cdot k_{e}\cdot s}{R+Ls}\cdot \theta_{g}\left(s\right)+J_{g}\cdot s^{2}\cdot \theta_{g}\left(s\right)$$

Considering that the inductance *L* of the generator is very small compared to the resistance *R*, the influence of the generator inductance on the dynamics of the generator can be ignored, so Equation 21 can be transformed into

(Equation 22) $$T_{g}\left(s\right)=\frac{k_{t}\cdot k_{e}\cdot s}{R}\cdot \theta_{g}\left(s\right)+J_{g}\cdot s^{2}\cdot \theta_{g}\left(s\right)$$

By applying inverse Laplace transform to Equation 22, the above equation can be expressed as

(Equation 23) $$T_{g}=\frac{k_{t}\cdot k_{e}}{R}\cdot \frac{d\theta_{g}}{dt}+J_{g}\cdot \frac{d^{2}\theta_{g}}{dt^{2}}$$

Therefore, the DC generator can be regarded as a mass-spring-damping system driven by the external excitation *T*_*g(t)*_, where the spring stiffness is zero, the equivalent mass is the rotational inertia of the generator, and the equivalent torsional damping coefficient is $\frac{k_{t}\cdot k_{e}}{R}$.

Vibro-electric coupling model of energy harvesting system under free vibration

This section combines the input module, the motion rectification module and the output module to carry out the electromechanical coupling dynamics analysis of the entire energy harvesting system. Since *T*_*z1*_ in the vibration input module is equivalent to the resistance torque *T*_*dzcl-A*_ acting on the transmission shaft by the large bevel gear (taking A as an example), *T*_*z1*_ can be expressed as

(Equation 24) $$T_{z1}=T_{qd}-J_{cdz}\cdot \alpha_{cdz}$$

Substituting Equation 15 into Equation 24 can obtain

(Equation 25) $$T_{z1}=\left(2\cdot J_{dzcl}+{k_{z}}^{2}\cdot J_{xzcl}\right)\cdot \frac{d^{2}x_{ct}}{r\cdot dt^{2}}+k_{z}\cdot T_{z2}$$

Substituting Equation 9 into Equation 25 can obtain

(Equation 26) $$F=\left(m_{ct}+\frac{J_{cl}+J_{cdz}}{r^{2}}\right)\cdot \frac{d^{2}x_{ct}}{dt^{2}}+\frac{\left(2\cdot J_{dzcl}+{k_{z}}^{2}\cdot J_{xzcl}\right)\cdot \frac{d^{2}x_{ct}}{r\cdot dt^{2}}+k_{z}\cdot T_{z2}}{r}=\left(m_{ct}+\frac{J_{cl}+J_{cdz}+2\cdot J_{dzcl}+{k_{z}}^{2}\cdot J_{xzcl}}{r^{2}}\right)\cdot \frac{d^{2}x_{ct}}{dt^{2}}+\frac{k_{z}\cdot T_{z2}}{r}$$

Since the small bevel gear is coaxially connected to the generator input shaft, *T*_*z2*_ is equivalent to the torque *T*_*g*_, and $\theta_{g}$ is equivalent to $\theta_{xzcl}$. Substituting Equation 23 into Equation 26 can get

(Equation 27) $$F=\left(m_{ct}+\frac{J_{cl}+J_{cdz}+2\cdot J_{dzcl}+{k_{z}}^{2}\cdot J_{xzcl}}{r^{2}}\right)\cdot \frac{d^{2}x_{ct}}{dt^{2}}+\frac{k_{z}\cdot \left(\frac{k_{t}\cdot k_{e}}{R}\cdot \frac{d\theta_{g}}{dt}+J_{g}\cdot \frac{d^{2}\theta_{g}}{dt^{2}}\right)}{r}=\left[m_{ct}+\frac{J_{cl}+J_{cdz}+2\cdot J_{dzcl}+{k_{z}}^{2}\cdot \left(J_{xzcl}+J_{g}\right)}{r^{2}}\right]\cdot \frac{d^{2}x_{ct}}{dt^{2}}+\frac{{k_{z}}^{2}\cdot k_{t}\cdot k_{e}}{R\cdot r^{2}}\cdot \frac{dx_{ct}}{dt}$$

After introducing the spring and vibration input plate parameters, the dynamics of the entire energy harvesting system is

(Equation 28) $$F=\left[m_{ct}+m_{srb}+\frac{J_{cl}+J_{cdz}+2\cdot J_{dzcl}+{k_{z}}^{2}\cdot \left(J_{xzcl}+J_{g}\right)}{r^{2}}\right]\cdot \frac{d^{2}x_{ct}}{dt^{2}}+\frac{{k_{z}}^{2}\cdot k_{t}\cdot k_{e}}{R\cdot r^{2}}\cdot \frac{dx_{ct}}{dt}+4\cdot k_{s}\cdot x_{ct}$$

where *m*_*srb*_ is the mass of the vibration input plate, and *k*_*s*_ is the stiffness coefficient of the spring. It can be seen from Equation 28 that the RE-VEH is a standard mass-spring-damping system. The damping part includes electrical damping and mechanical damping. The main components of mechanical damping are inter-tooth friction and bearing friction. The inter-tooth friction and bearing friction are very small under ideal lubrication conditions, so the mechanical damping is neglected in this paper. Therefore, the equivalent mass *m*_*eq*_, equivalent damping coefficient *c*_*eq*_ and equivalent stiffness *k*_*eq*_ of the system can be expressed as

(Equation 29) $$\left\{ \begin{array}{l} m_{eq}=m_{ct}+m_{srb}+\frac{J_{cl}+J_{cdz}+2\cdot J_{dzcl}+{k_{z}}^{2}\cdot \left(J_{xzcl}+J_{g}\right)}{r^{2}}\\ c_{eq}=\frac{{k_{z}}^{2}\cdot k_{t}\cdot k_{e}}{R\cdot r^{2}}\\ k_{eq}=4\cdot k_{s} \end{array}\right.$$

Substituting Equation 29 into Equation 28, the dynamics of the vibration energy harvesting system can be rewritten as

(Equation 30) $$F=m_{eq}\cdot \frac{d^{2}x_{ct}}{dt^{2}}+c_{eq}\cdot \frac{dx_{ct}}{dt}+k_{eq}\cdot x_{ct}$$

The vibration-electric coupling dynamics of the entire RE-VEH system can be expressed as

(Equation 31) $$\left\{ \begin{array}{l} F=m_{eq}\cdot \frac{d^{2}x_{ct}}{dt^{2}}+c_{eq}\cdot \frac{dx_{ct}}{dt}+k_{eq}\cdot x_{ct}\\ U_{emf}=\frac{k_{e}\cdot k_{z}}{r}\cdot \frac{dx_{ct}}{dt} \end{array}\right.$$

(Equation 32) $$x=Ae^{-\varsigma p_{n}t}\;\sin\left(p_{d}t+\varphi \right)$$

where *x*, *A*, ς, *p*_*n*_, *p*_*d*_ and φ are the transient displacement, vibration amplitude, damping ratio, natural circular frequency and phase of the vibrating system, respectively.

Combining Equation 31 and Equation 32, the vibro-electric coupling model of the RE-VEH under free vibration mode can be described as

(Equation 33) $$\left\{ \begin{array}{l} 0=m_{eq}\cdot \frac{d^{2}x}{dt^{2}}+c_{eq}\cdot \frac{dx}{dt}+k_{eq}\cdot x\\ x=Ae^{-\varsigma p_{n}t}\;\sin\left(p_{d}t+\varphi \right)\\ U_{emf}=\frac{k_{e}\cdot k_{z}}{r}\cdot \frac{dx}{dt} \end{array}\right.$$

### Quantification and statistical analysis

Microsoft Visio 2019 is used to generate the visual images in the manuscript. The voltage signals are captured by the DS1102Z-E digital oscilloscope. The force-displacement signals are captured by the force sensor and displacement sensors integrated into the Landmark 370 servo-hydraulic test system at a sampling frequency of 300 Hz. MATLAB 2022a is used to process experimental data and generate visual images in the manuscript. Through MATLAB 2022a, the voltage signal and force-displacement data are processed to analyze the input and output characteristics of the system.

## Data Availability

• The attached supplemental information file includes all dataset generated or analyzed during this study. • This paper does not report original code. • Any additional information is available from the lead contact upon request.
