# Supplementary material for: Parameter optimization analysis of rotary electromagnetic vibration energy harvester for performance enhancement under free vibration
Source: iScience. 2023 Sep 21;26(10):107989. doi: 10.1016/j.isci.2023.107989 (PMC10558786; doi:10.1016/j.isci.2023.107989)
Supplement: Document S1. Tables S1 and S2 [file mmc1.pdf]

iScience, Volume *26*

Supplemental information

Parameter optimization analysis of rotary electromagnetic vibration energy harvester for performance enhancement under free vibration

Juhuang Song, Fugui Zhang, Lingfei Qi, Hao Cao, Yuan Wang, Zutao Zhang, and Jinyue Yan

Table S1. Abbreviation and its explanation, related to STAR Methods.

Abbreviation	Explanation	Unit
VEH	Vibration energy harvesting	\
RE-VEH	Rotary electromagnetic vibration energy harvesters	\
EMF	Electromotive force	\

Table S2. Symbols and its explanation, related to STAR Methods.

Symbols	Explanation	Unit
F	External excitation force	N
m_{ct}	Mass of the rack	kg
x_{ct}	Linear displacement of the rack	m
v_{ct}	Linear velocity of the rack	m/s
a_{ct}	Linear acceleration of the rack	m/s^2
J_{cl}	Moment of inertia of the gear	kg m^2
θ_{cl}	Angular displacement of the gear	rad
ω_{cl}	Angular velocity of the gear	rad/s
α_{cl}	Angular acceleration of the gear	rad/s^2
J_{cdz}	Moment of inertia of the transmission shaft	kg m^2
θ_{cdz}	Angular displacement of the transmission shaft	rad
ω_{cdz}	Angular velocity of the transmission shaft	rad/s
α_{cdz}	Angular acceleration of the transmission shaft	rad/s^2
J_{dzcl}	Moment of inertia of the large bevel gear	kg m^2
θ_{dzcl}	Angular displacement of the large bevel gear	rad
ω_{dzcl}	Angular velocity of the large bevel gear	rad/s
α_{dzcl}	Angular acceleration of the large bevel gear	rad/s^2
J_{xzcl}	Moment of inertia of the small bevel gear	kg m^2
θ_{xzcl}	Angular displacement of the small bevel gear	rad
ω_{xzcl}	Angular velocity of the small bevel gear	rad/s
α_{xzcl}	Angular acceleration of the small bevel gear	rad/s^2
T_{z1}	Resistance torque acting on the transmission shaft	N m
T_{qd}	Driving torque acting on the transmission shaft	N m
T_{cdz}	Torque acting on the gear by the transmission shaft	N m
T_{z2}	Resistance torque acting on the small bevel gear	N m
r	Radius of the gear	m
$T_{dzcl\text{-}A}$	Resistance torque acting on the transmission shaft by the large bevel gear A	N m
k_z	Transmission ratio between the large bevel gear and the small bevel gear	\
T_{xzcl}	Driving torque obtained by the small bevel gear	N m
$T_{dzcl\text{-}B}$	Driving torque obtained by the large bevel gear B	N m
U_{emf}	Induced electromotive force of the generator	V
T_g	Input torque of the generator	N m
r_i	Generator internal resistance	Ω
L	Generator inductance	H
R_e	External resistance	Ω

Table S2. Symbols and its explanation, related to STAR Methods.

Symbols	Explanation	Unit
k_e	Generator induced electromotive force constant	\
k_t	Generator torque constant	\
ω_g	Rotational angular velocity of generator	rad/s
ω_e	Generator phase change angular velocity	rad/s
i	Loop current	A
T_i	Resistance torque generated by the three-phase alternator	N·m
J_g	Moment of inertia of the generator	kg·m ^{2}
θ_g	Rotation angle of the generator shaft	rad
R	Sum of generator internal resistance and external resistance	Ω
k_s	Spring stiffness	N/m
m_{srb}	Mass of vibration receiver	kg
m_{eq}	System equivalent mass	kg
c_{eq}	System equivalent damping coefficient	N/(m/s)
k_{eq}	System equivalent stiffness	N/m
F_s	Harmonic excitation	N
H	Vibration amplitude	m
ω	Vibration circular frequency	rad/s
m	Mass of the standard vibration system	kg
c	Damping coefficient of the standard vibration system	N/(m/s)
k	Stiffness of the standard vibration system	N/m
x	Transient displacement of the standard vibration system	m
x_1	Transient solution of the standard vibration system	m
x_2	Steady-state solution of the standard vibration system	m
X	Amplitude of the simple harmonic function	m
φ	Phase angle of the simple harmonic function	rad
ζ	Damping ratio of the standard vibration system	\
p_n	Natural circular frequency of the standard vibration system	rad/s
